# Anopheles Midgut Epithelium Evades Human Complement Activity by Capturing Factor H from the Blood Meal

**DOI:** 10.1371/journal.pntd.0003513

**Published:** 2015-02-13

**Authors:** Ayman Khattab, Marta Barroso, Tiera Miettinen, Seppo Meri

**Affiliations:** 1 Research Program Unit, Immunobiology Research Program, Haartman Institute, University of Helsinki, Helsinki, Finland; 2 Helsinki University Central Hospital, Helsinki, Finland; National Institute of Allergy and Infectious Diseases, UNITED STATES

## Abstract

Hematophagous vectors strictly require ingesting blood from their hosts to complete their life cycles. Exposure of the alimentary canal of these vectors to the host immune effectors necessitates efficient counteractive measures by hematophagous vectors. The *Anopheles* mosquito transmitting the malaria parasite is an example of hematophagous vectors that within seconds can ingest human blood double its weight. The innate immune defense mechanisms, like the complement system, in the human blood should thereby immediately react against foreign cells in the mosquito midgut. A prerequisite for complement activation is that the target cells lack complement regulators on their surfaces. In this work, we analyzed whether human complement is active in the mosquito midgut, and how the mosquito midgut cells protect themselves against complement attack. We found that complement remained active for a considerable time and was able to kill microbes within the mosquito midgut. However, the *Anopheles* mosquito midgut cells were not injured. These cells were found to protect themselves by capturing factor H, the main soluble inhibitor of the alternative complement pathway. Factor H inhibited complement on the midgut cells by promoting inactivation of C3b to iC3b and preventing the activity of the alternative pathway amplification C3 convertase enzyme. An interference of the FH regulatory activity by monoclonal antibodies, carried to the midgut via blood, resulted in increased mosquito mortality and reduced fecundity. By using a ligand blotting assay, a putative mosquito midgut FH receptor could be detected. Thereby, we have identified a novel mechanism whereby mosquitoes can tolerate human blood.

## Author Summary

Mosquitoes are important vectors in the transmission of many human diseases. Their life cycle requires a blood meal to be completed. Ingested blood contains bioactive molecules belonging to the innate immune defense mechanisms against microbes, like the complement system, that can damage foreign cells. We have identified in this study a mechanism whereby mosquitoes can escape the damaging activity of the complement system in the ingested human blood. The mosquito midgut epithelial cell surface captured factor H, a natural regulator of the alternative pathway of complement activation, from the ingested blood. Consequently, the deposition of C3b, a key complement component, on the epithelial cell surface was impaired and cell death was avoided. Interfering with the complement regulatory activity of factor H by monoclonal antibodies, carried to the midgut via blood feeding, increased mosquito mortality and reduced fecundity. The putative *Anopheles* mosquito factor H binding proteins could be transmission blocking vaccine candidates targeting the malaria parasite carrying vectors.

## Introduction

Mosquitoes can transmit important parasitic diseases such as malaria and filariasis and viral diseases such as yellow fever, dengue, Rift Valley fever and the West Nile virus. *Anopheles*, *Aedes*, *Culex*, *Coquillettidia*, *Mansonia* and *Ochlerotatus* species are the best known disease transmitting mosquitoes[1]. They all require a blood meal to obtain proteins from their hosts. Blood proteins are needed for the development and laying of eggs to complete the life cycles of the mosquitoes. Parasites and viruses carried in the host blood can therefore be transmitted to other individuals of the same host species and sometimes also to other species if the organisms can multiply inside mosquitoes and survive in the new hosts. Ingestion of host blood has been suggested to pose a danger to mosquitoes as a result of exposing the alimentary canal (AC) to bioactive molecules that normally exist in host blood as part of the host defense mechanisms against microbes. Likewise, other ingested blood-derived factors such as antibodies, hemoglobin-derived peptides, enzymes and signaling molecules could alter the physiology of hematophagous vectors (reviewed in[2]). The most immediate system that has been shown to be overcome by mosquitoes and other hematophagous vectors is the coagulation system[3]. Mosquitoes’ and ticks’ salivary molecules were found to inhibit blood clotting at the biting site. The injected saliva contained anti-coagulants that permitted smooth flow of blood from the skin of the host to the vector and prevented blockage of the blood sucking capillary[3].

The complement system is a host defense mechanism that could impose danger to disease vectors upon blood feeding. It is a cascade that attacks the surfaces of foreign cells[4]. Complement plays a central role in the innate immune response to combat microbial infections. There are three pathways to activate complement, the classical, the alternative and the lectin pathway. The classical pathway is triggered when C1 interacts with antibodies bound to their antigens. This results in the cleavage of C4 and C2 and the formation of the classical pathway C3-convertase, C4b2a, which cleaves C3 into C3b. The lectin pathway is activated when the mannan-binding lectin (MBL) or one of the three ficolins binds to sugar residues (Man, GalNAc or acetylated sugars) on target surfaces. The alternative pathway is initiated through a spontaneous cleavage of an internal thioester bond in C3. All three pathways converge in the cleavage of the C3 protein. This will direct phagocytosis of targets and leads ultimately to the formation of membrane attack complexes (MAC). MAC is a pore that can cause damage or lysis of the target cells.

Under normal circumstances, activation of complement is kept under tight control by the coordinated action of soluble (e.g. C1-INH, C4bp, factor H, factor I, S-protein and clusterin) and membrane-associated (DAF, CR1, MCP, CRIg and CD59) complement regulatory proteins[5]. The control of complement activity involves inhibition of assembly or dissociation of C3 convertases and the inhibition of the membrane attack complex formation[5]. Among the complement regulators, factor H (FH) plays an integral role in controlling complement activation. It regulates the positive feedback loop of the alternative pathway, which could otherwise lead to excessive activation of C3 and damage to nearby cells [6,7]. FH regulates complement activation on self-cells surfaces by possessing both cofactor activity for the factor I-mediated C3b cleavage, and decay accelerating activity to dissociate the C3-convertase, C3bBb. FH protects self surfaces because it binds to glycosaminoglycans (GAGs) and other polyanions that are generally present on host cell surfaces. Microbes usually lack these structures [6,7].

Given the importance of FH in controlling complement activation, several pathogens that are known to resist complement-mediated killing have been shown to express FH-binding proteins on their surfaces [5]. We hypothesized that this strategy could be used by the hematophagous vectors, as well, to avoid complement-mediated damage when exposed to blood. We therefore analyzed how complement is regulated in the midgut after a blood meal. We observed that complement remained active and could become activated in the mosquito midgut. This suggested that an evasion mechanism to avoid the detrimental consequences of complement activation must exist in the mosquito midgut cells. Accordingly, FH was found to bind to the luminal surface of the mosquito proventriculus (located between the esophagus and anterior midgut), the anterior midgut and the anterior-posterior region of the midgut. Complement activation on these surfaces was shown to be prevented. Taken together, complement activation was shown to occur in the mosquito midgut but the *Anopheles* mosquitoes were able to protect themselves at this critical site via a novel evasion mechanism.

## Materials and Methods

### Mosquito rearing

*Anopheles stephensi* (Nijmegen strain) and *Anopheles gambiae* 4ARR (MR4 strain) adult mosquitoes were maintained in 20x20x20 cm gauze cages at 28°C, 80 ± 5% relative humidity, and a photo-scotophase of 12:12 light:dark. The mosquitoes had access to a 5% sucrose solution on a cotton pad. The larvae were reared in tap water on plastic trays and fed daily with Tetramin fish food. Pupae were collected daily and placed in adult cages for emergence. Adult mosquitoes were regularly fed on a blood meal that contained 1:1 human erythrocytes and normal human serum (NHS) using the glass membrane feeder for maintaining the mosquito rearing cycle.

### Alimentary canal (AC) and midgut dissection

Cold-anesthetized mosquitoes were dissected over a sterile glass slide containing a drop of PBS under a stereomicroscope. ACs (proventriculus, anterior and posterior midgut) or midguts (anterior and posterior midgut) were isolated and placed in PBS, LB-medium, PBS-EDTA or in a commercial ELISA dilution buffer.

### Measurement of complement activation and residual complement activity (ELISA)

To measure the extent of complement activation in the mosquito midguts post blood feeding (PBF), *A. stephensi* mosquitoes were allowed to feed on a human volunteer arm and midguts were dissected from a pair of mosquitoes at 10, 30 and 90 min PBF. Each isolated pair of mosquitoes was placed in 200 μl specimen diluent from the MicroVue C3a Plus EIA kit, macerated for 30 sec with the tip of 21G sterile needle and centrifuged at 16,000*g* for 5 min at 4°C. Supernatants were immediately frozen at -70°C until processing. The average serum content in the midgut of a BF mosquito was estimated to be about 1 μl, this value was used when estimating specimen dilution for C3a and C5b-9 ELISAs. C3a concentration (ng/ml) was measured using the MicroVue C3a Plus EIA kit according the manufacturer’s instructions. C5b-9 concentration (AU/ml) was measured as previously described[8].

### Viability and serum sensitivity of midgut microbiota

To study the effect of complement in the mosquito midgut on bacterial counts (viability) PBF, *A. stephensi* mosquitoes were allowed to feed on a blood meal that contained 1:1 human erythrocytes and either NHS or HIS. At 30, 90 and 24 h PBF, 5 mosquitoes representing each condition and time point were dissected and the isolated midguts were placed in 1 ml of LB-medium. The midguts were then macerated with 21G sterile needles and the sample tubes were vortexed for 30 sec. The standard plate counting method that involves serial dilutions, plating and counting of bacterial colonies was used under aerobic conditions to determine the number of live bacteria per midgut. Midguts from 5 sugar-fed mosquitoes were treated similarly to estimate the initial microbial load before blood feeding. A bacterial colony morphologically representing the majority of the colonies growing from either feeding on NHS or HIS based blood meal was isolated and analyzed for serum sensitivity, when incubated in 20% NHS or HIS for 1 h as previously described[9]. The identities of both bacterial strains were assessed by 16S ribosomal DNA PCR as previously described[10].

### Mosquito sections and immunofluorescence staining

*A. stephensi* and *A. gambiae* mosquitoes were allowed to feed on a human volunteer arm. *A. stephensi* female mosquitoes were also fed on a blood meal that contained normal mouse serum (containing active complement) and mouse erythrocytes in a membrane glass feeder and blood-fed mosquitoes were left to rest for 2 h PBF. Blood- and sugar-fed (BF and SF) mosquitoes were then cold-anesthetized and placed in PBS, pH 7.4, containing 4% paraformaldehyde for 2 h followed by the dehydration and the clearing steps prior to embedding in paraffin wax. Each block was cut into 4-μm thick sagittal sections and representative slides were stained with HE to check the quality of the mosquito sections. Prior to immunofluorescence staining, the sections were dewaxed in xylene 3x5 min, rehydrated in a series of ethanol baths (100, 95 and 70%) for 3 min each and rinsed twice in distilled H_2_O for 5 min each. Dewaxed slides made from BF and SF mosquitoes were then treated with 1% BSA-PBS for 30 min to prevent nonspecific protein binding. BSA-PBS was then replaced by 1:500 dilution of goat polyclonal anti-human FH (A312, Quidel, San Diego), rabbit polyclonal anti-human C3c (A0062, Dako), goat polyclonal anti-human C5 (A306, Quidel, San Diego) or murine monoclonal anti-human SC5b-9 (A239, Quidel, San Diego) antibodies. After a 1-hour incubation at room temperature (RT) in a humidified chamber, the slides were washed 3x10 min in PBS-0.05% Tween 20 (PBS-T). Binding of primary antibodies was then detected by overlying the slides with 1:1000 dilution of Alexa Fluor 488 Donkey anti-goat IgG, goat anti-rabbit IgG or goat anti-mouse IgG (Invitrogen), respectively. Cell nuclei were stained with 30 nM DAPI (4′,6-diamidino-2-phenylindole) included in the diluted secondary antibody. Following 1 h of incubation in a dark humidified chamber at RT, slides were washed 3x10 min in PBS-T. Cover slips were mounted on slides using Mowiol-based antifading medium and kept at 4°C for at least 30 min before examining with the Olympus BX51 fluorescence microscope. Images were captured using the Olympus DP70 camera with the help of DP controller software. Slides of sugar-fed mosquitoes were treated similarly and served as negative controls for binding of the primary antibodies to mosquito proteins in the immunofluorescence assays. For colocalizing bound FH with carbohydrates present on the surface of the midgut epithelium, Alexa Fluor 594 conjugated concanavalin-A (25 ug/ml) was combined with Alexa Fluor 488 Donkey anti-goat IgG that detects binding of the primary antibody to FH. Following 1 h of incubation in a dark humidified chamber at RT, slides were washed 3x5 min in PBS-T. Cover slips were mounted on slides using Mowiol-based antifading medium and kept at 4°C for at least 30 min before examining with Leica TCS CARS SP8 confocal microscope.

### Terminal deoxynucleotidyl transferase mediated dUTP nick end labeling (TUNEL) assay

DNA fragmentation, possibly indicative of apoptosis or cell death, was studied using fluorometric TUNEL kit (TACS 2 TdT-Fluor In Situ Apoptosis Detection Kit, TREVIGEN, Gaithersberg). Briefly, *A. stephensi* mosquito sections on dewaxed slides were treated with proteinase K for 15 min at RT, washed 2x in deionized water for 2 min each and immersed in labeling buffer for 5 min. Sections were then covered with the labeling reaction containing TdT dNTP, Mn2+, TdT enzyme and labeling buffer and incubated at 37 C for 1h. Labeling reaction was then stopped in stop buffer for 5 min and the slides were washed 2x in deionized water for 5 min each at RT. Sections were then covered with Strep-Alexa 488 conjugate solution and incubated for 20 min at RT followed by washing 2x in PBS for 2 min each and mounted afterwards with Mowiol mounting medium. TUNEL-positive mosquito section slides were prepared by treating dewaxed slides with TACS-Nuclease to induce DNA fragmentation. After 30 min incubation at RT slides were washed 2x in PBS for 2 min each. Induced DNA fragmentation was then detected as previously described.

### Mosquito survival and fecundity assays

Blood meals that contained 1 mg/ml monoclonal anti-human FH antibody (131X) and 1:1.1 human erythrocytes and either NHS or HIS were pre-incubated at RT for 20 min prior to *A. stephensi* mosquito feeding. Mosquitoes were also fed on NHS and erythrocytes in the same ratio in a third experimental condition. Cold-anesthetized blood-fed mosquitoes were separated and placed in new cages. Dead mosquitoes were counted daily for 7 days PBF. On day 8, egg collectors were placed inside mosquito cages and the laid eggs were counted the following day. The average number of blood-fed mosquitoes from 3 biological repetitions of NHS+131X, HIS+131X and NHS test conditions was 31.8±11.3, 34.5±7.6 and 31.7±7.6, respectively. In a fourth experiment 10 blood-fed mosquitoes from each test condition were collected 2 h PBF and placed in 4% paraformaldehyde for 2 h followed by the dehydration and the clearing steps prior to embedding in paraffin wax. Sagittal sections were then prepared as described above and analyzed for the presence of apoptotic/dead cells using the TUNEL assay described above and fluorescence microscopy.

### Western blot analysis

The kinetics of complement C3 activation/degradation in the mosquito midgut following blood feeding was assessed by Western blot analysis. A. stephensi mosquitoes were allowed to feed on a human volunteer arm and midguts were dissected at 10, 20, 30 and 90 min from a single mosquito for each time point. Individual midguts were collected in 200 μl of PBS+10 mM EDTA and macerated for 30 sec with 21G needle and centrifuged at 16,000*g* for 5 min at 4°C. Supernatants diluted 1:1 with a reducing SDS-PAGE sample buffer were run on 10% SDS-PAGE gels, and separated proteins were transferred to a nitrocellulose membrane and probed with 1:5000 dilution of rabbit polyclonal anti-human C3c. Binding of the primary antibody was visualized by incubating the membrane with 1:20,000 dilution of goat anti-rabbit IgG coupled to HRP (NEF812, Perkin Elmer life Sciences, Inc.). The blot was then developed by the enhanced chemiluminescent (ECL) Western blot analysis system-based detection according to the manufacturer’s instructions (GE Healthcare, Life Sciences, Bucks, UK) followed by exposure to Super RX film (Fujifilm). Possible deposition of C3b or the presence of its inactivation products, iC3b or C3d, on the luminal surface of the proventriculus and the midgut epithelium was similarly investigated using ACs isolated from 6 mosquitoes that were fed on NHS+human erythrocytes using the glass membrane feeder. ACs were macerated in PBS+protease inhibitor cocktail (Roche) using a 21G needle and washed 4x with PBS-T that contained the protease inhibitor cocktail. After a wash with PBS the AC tissues were pelleted by centrifugation at 16,000*g* for 5 min at 4°C. Proteins were then extracted with 90 μl 1x reducing SDS-PAGE sample buffer and a volume equivalent to 1 AC was run on a gel for C3b, iC3b, C3c or C3d detection. An equivalent number of mosquitoes that were normally fed on 5% sucrose was treated in parallel and served as a control. Binding of FH to the AC epithelium was assessed similarly using the former sample set with 1:5000 dilution of goat polyclonal anti-human FH as the primary WB antibody and a 1:20,000 dilution of rabbit anti-goat coupled to HRP (Dako, P0160) as the secondary antibody. Loading control lanes were included in WB analysis, when appropriate. They were probed with the MRA-258 monoclonal antibody (MR4, the BEI Resources Repository, NIAID, NIH) that recognizes ≈150 kDa protein in the mosquito midgut extract.

### Ligand blot analysis

Possible FH binding protein(s) in the midgut membrane extract were detected by using the ligand blot analysis[11]. Briefly, membrane proteins were extracted by incubating the *A. stephensi* mosquito midguts in the membrane extraction buffer (containing the protein inhibitor cocktail) from the ProteoJET membrane protein extraction kit (Fermentas, K0321) for 2 h at 4°C with constant shaking. Extracted membrane proteins were recovered by centrifugation at 16,000*g* for 15 min at 4°C. A volume equivalent to 1 midgut per lane was then loaded onto 10% SDS-PAGE gel under non-reducing conditions. Resolved membrane proteins were transferred to nitrocellulose membranes and overlaid after a blocking step with 3% non-fat milk for 1h with either 20% HIS (a source of FH) or PBS (a negative control for FH binding) and incubated overnight at 4°C. After 5 washes in PBS-T, the membranes overlaid earlier with HIS were further incubated in either PBS (a negative control for binding of the secondary antibody) or with 2 μg/ml of the monoclonal anti-human FH antibody (131X). After a 1-hour incubation at RT and 5 washes in PBS-T, the membranes were incubated in a 1:10,000 dilution of goat anti-mouse IgG coupled to HRP (NFH822, Perkin Elmer life Sciences, Inc.). After a 1-hour incubation at RT and 5 washes in PBS-T, the blots were developed as described above.

### Statistical analysis

Data were analyzed with JMP 11 (SAS Institute Inc.). Paired Student’s t-test was used to calculate the statistical significance of complement activation on the bacterial load in the mosquitoes midgut and the in-vitro serum sensitivity of midgut bacteria and of blocking of FH activity with antibodies on egg count. To calculate the statistical significance of blocking FH activity with antibodies on mosquito survival, life tables were constructed for each experimental condition, and survival curves were analyzed by using the Kaplan-Meier log-rank analysis. Asterisks in figures indicate the different p values: *, p < 0.05; **, p < 0.01; ***, p < 0.001. All experiments were repeated at least three times. Results from different repetitions of experiments were pooled together and are presented as the mean and, when appropriate, representative images are shown. In all figures, error bars depict standard errors of the mean.

## Results

### Complement activity in the mosquito midgut

**Fig 1:**
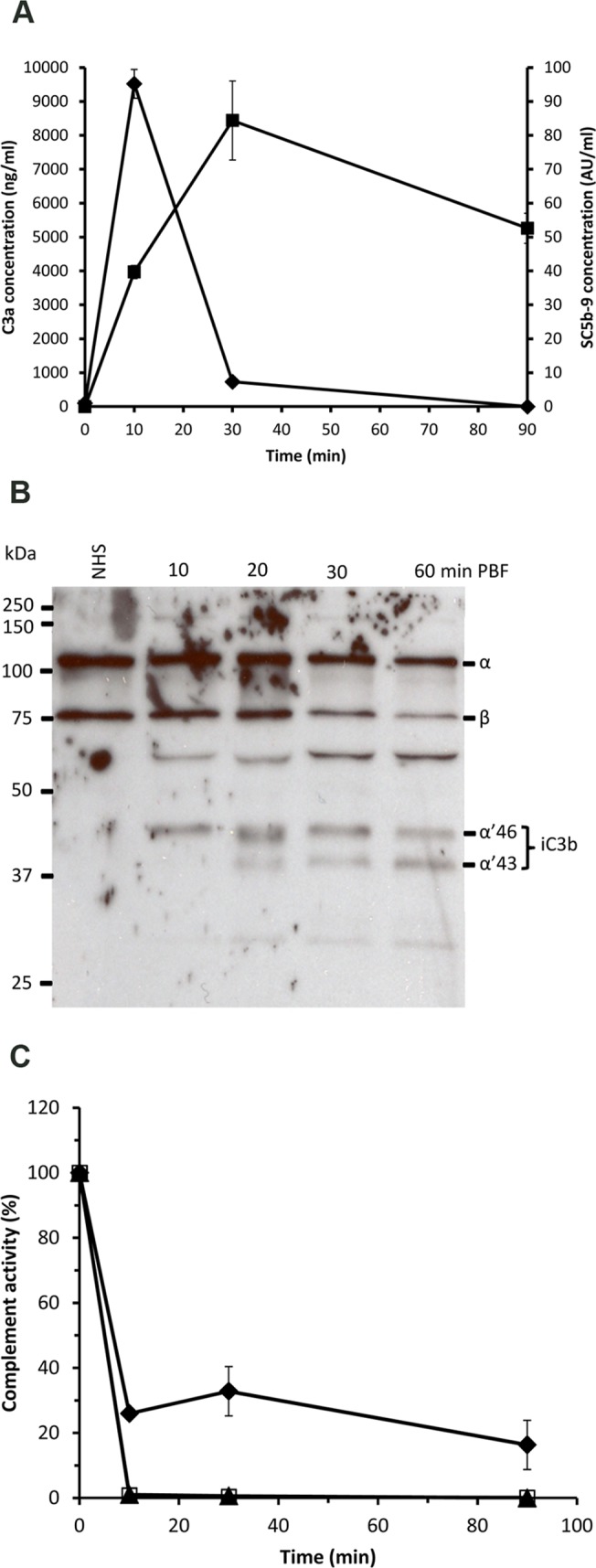
Complement activation in the *A. stephensi* mosquito midgut after feeding on blood from the arm of a human volunteer. (A) The concentrations of the complement activation products C3a (♦) and SC5b-9 (■) were measured in sera collected from mosquito midguts at 10, 30 and 90 min PBF on a human volunteer arm. C3a and C5b-9 were also measured in a serum sample (zero time) of the volunteer. C3a level peaked at 10 min PBF and then dropped sharply at 30 min PBF, whereas the C5b-9 level peak was reached later at 30 min PBF. (B) Conversion of C3 into iC3b as a sign of regulated complement activation was assessed by Western blot analysis. Serum samples taken before blood feeding and collected from mosquito midgut at 10, 20, 30 and 90 min PBF showed decreasing intensities of C3 α-chain and increasing intensities of α’ 46 and α’43 kDa chains (iC3b fragments) of over time. (C) Residual complement activity in serum samples collected from mosquito midguts at 10, 30 and 90 min PBF. Both the alternative (AP; ▲) and the lectin pathways (LP; □) of complement activation appeared to be completely inhibited or consumed at 10 min PBF, whereas about 20% of the classical pathway (CP; ♦) activity was still detectable at 90 min PBF.

In the current study we tested whether components of the complement system in the blood ingested by *Anopheles* mosquitoes could injure midgut cells, and if not so, how do the midgut cells manage to survive the potent cytotoxic activity of complement? We first tested whether the human complement system in the ingested blood becomes activated in the mosquito midgut. Therefore, *A. stephensi* mosquitoes were allowed to feed on a human volunteer arm. This was followed by measuring the concentrations of the anaphylatoxin C3a and the soluble terminal complement complex (TCC, also known as SC5b-9) as indicators of complement activation. For this, the blood bolus serum samples were isolated from BF mosquito midguts. As shown in Fig. 1A the complement system became strongly activated in the mosquito midgut. The peak level of activation was found 10 min PBF as shown by a 100-fold increase in the concentration of C3a, when compared to the basal level (zero time) in the human volunteer serum (Fig. 1A). Moreover, the C3a level dropped to only 7-fold at 30 min PBF and reached the basal level at 90 min PBF (Fig. 1A). A more drastic indication of complement activation was observed, when the SC5b-9 level was measured in the bolus serum samples. The SC5b-9 concentration increased to 150-fold at 30 min PBF, when compared to the basal level in the volunteer serum sample (Fig. 1A). SC5b-9 level went down to 100-fold at 90 min PBF (Fig. 1A). Both C3a and SC5b-9 measurements in blood bolus serum samples indicated that complement was strongly activated in the mosquito midgut. The different kinetics of the two complement activation products are expected because they are raised at different steps of complement activation and, as a multimolecular complex SC5b-9 takes a longer time to form. The clearance rates of C3a and SC5b-9 are also different, C3a being more rapidly cleared.

To support the assumption that C3a was produced as a result of complement activation we analyzed whether C3 was converted into C3b and further to iC3b in the same sample set by Western blotting (WB) analysis. The WB data showed that more than 50% of the initial C3 had become converted to iC3b in the mosquito midgut 60 min PBF (Fig. 1B). This indicated that C3 had become activated to C3a and C3b and C3b was further inactivated to iC3b. Consequently, we also measured the residual complement activity that remained in the mosquito midgut at 10, 30 and 90 min PBF. The results showed a complete loss of the alternative and lectin pathway activities in just 10 min PBF (Fig. 1C). At the same time point about 74% of the classical pathway activity was lost. At 90 min approximately 20% of classical pathway activity could be still detected (Fig. 1C). Since the residual complement activity measurements for the classical pathway were based on quantifying the levels of SC5b-9 resulting from activating complement in the test samples, some active C3 must have been remained in the samples. Taken together, these results indicated that the complement system in human blood became activated in the fluid phase in the mosquito midgut.

### Complement-mediated killing of mosquito midgut microbiota

**Fig 2:**
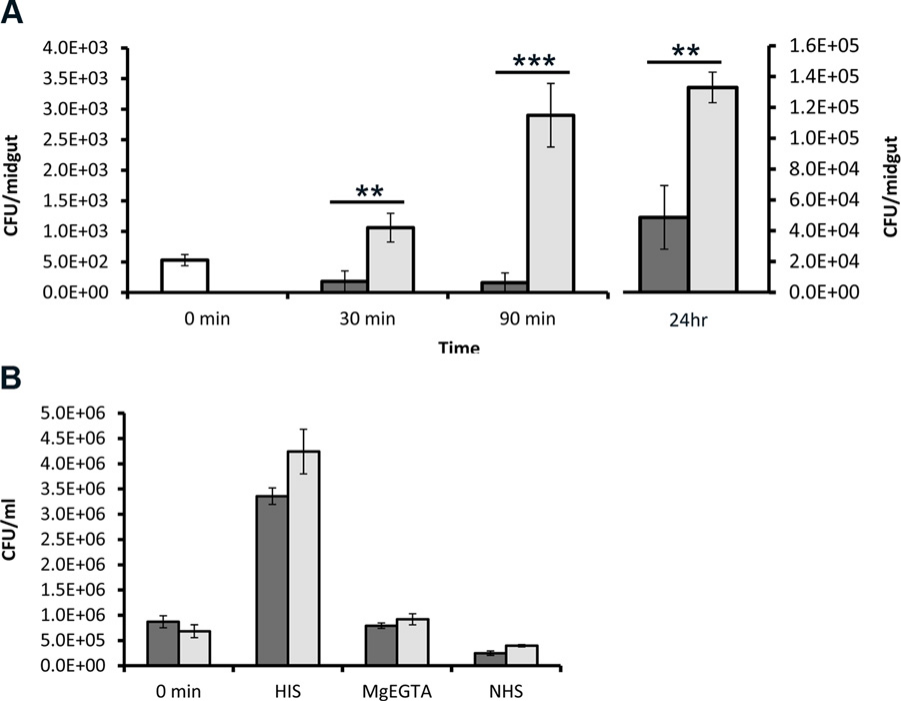
Complement-mediated killing of bacteria in the *A. stephensi* mosquito midgut. (A) Standard plate counting of bacteria isolated from whole midgut contents of mosquitoes. Mosquitoes that were fed on a NHS-containing blood meal (black bars) showed significantly lower levels of bacterial counts in the midguts than HIS-fed mosquitoes (grey bars; at 30 and 90 min PBF) or the sugar-fed mosquitoes (white bar, 0 min). Also, at the 24h time point significantly lower levels of bacteria in the midguts of NHS-fed mosquitoes were seen (note the different scale). Significantly higher levels of bacteria were also observed in HIS- and NHS-fed mosquitoes than in sugar-fed mosquitoes. (B) The major bacterial strains isolated from midguts of mosquitoes fed on NHS or HIS (*E. cloacae*) containing blood meal at 24 h PBF were tested for their resistance to complement-mediated killing in-vitro. The two strains grew equally well in HIS after a 1h incubation at 37°C. Also, both strains were killed equally well in MgEGTA containing serum and in NHS. In MgEGTA serum the classical pathway is blocked.

As the initial experiments showed that complement became strongly activated in the midguts of mosquitoes that were fed naturally with human blood, we next asked whether this activation would impose any threat to cells in the mosquito midgut. As a surrogate marker for the ability of complement to kill cells in the mosquito midgut we chose first to study effects on the midgut microbiota. Here, *A. stephensi* mosquitoes were allowed to feed artificially either on normal human serum (containing functional complement) or heat-inactivated serum (containing non-functional complement) in addition to human erythrocytes under both conditions. Mosquitoes were dissected at 30 min, 90 min and 24 h PBF and the viable bacterial contents of the midguts were estimated by a plate colony counting procedure. As expected, feeding mosquitoes on NHS decreased the bacterial count in midguts to 34% and 17% of what was recorded for mosquitoes fed on sugar or HIS, respectively, when dissection took place at 30 min PBF (Fig. 2A). At the same time point bacterial count showed two-fold increase in midguts of HIS-fed mosquitoes compared to sugar-fed mosquitoes. This simply shows that feeding on blood containing active complement reduces bacterial count, while feeding on non-active complement containing blood meal increased midgut bacterial load. At 90 min PBF the bacterial counts in midguts of mosquitoes fed on NHS dropped to 5% relative to feeding on HIS (Fig. 2A). At 24 h PBF the difference between bacterial counts in NHS and HIS feeding conditions was not drastic, but remained significant as the decrease in bacterial count in NHS was only 37% of what was recorded in HIS (Fig. 2A). The increasing number of bacterial count at the 24 h time point in midguts of mosquitoes fed on NHS relative to the former time points suggested that serum resistant bacterial strains or just the remaining surviving bacteria might have overgrown. Therefore, morphologically dominant colonies from both NHS and HIS conditions were selected and tested for their ability to resist complement-mediated killing. The bacteria were identified to belong to the genus *Enterobacter* (*E. cloacae*) in both cases. *Enterobacter* isolates from both conditions were, found to be sensitive to complement and behave similarly in their response to complement-mediating killing (Fig. 2B). Therefore, the likeliest explanation for bacterial overgrowth in midguts of mosquitoes fed on NHS after 24 h was the lack of sufficient complement activity to kill all midgut bacteria. Altogether, this data clearly shows that complement activation in the mosquito midgut has a detrimental effect on living cells in the midgut lumen.

### Binding of human FH to the mosquito midgut epithelium

**Fig 3:**
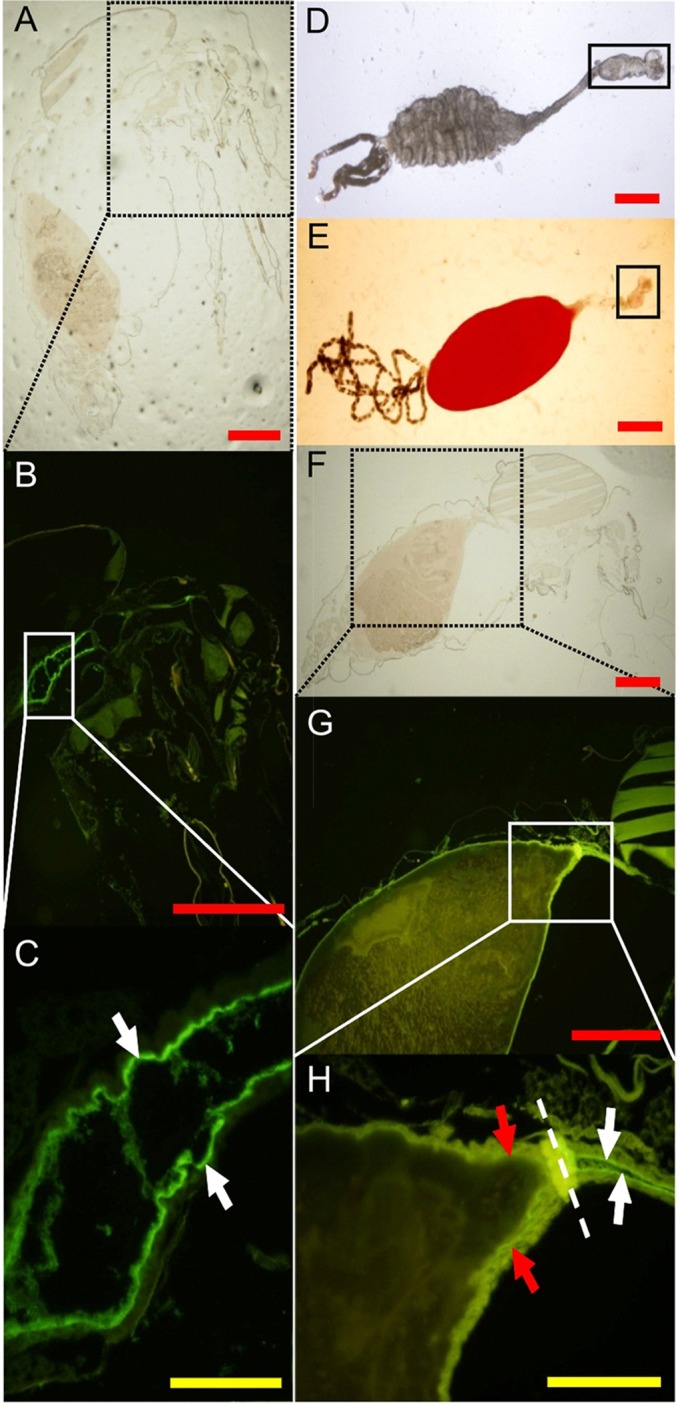
Binding of human FH to the mosquito proventriculus and midgut epithelial surface in indirect immunofluorescence assays. Female *A. gambiae* mosquitoes were fed with blood on a human volunteer’s arm and cut thereafter into sagittal sections. (A and F) Bright field microscopic images of whole mosquito sagittal sections used for the IF staining. Black-dotted squares show the locations of the fluorescently-labeled areas shown in B and G. (B and G) Binding of FH from the blood meal to the epithelial surface of the mosquito. AC is shown as a green fluorescence. White squares show the location of the proventriculus (magnified in C) and the anterior and the anterior-posterior midgut (magnified in H). (C) A higher magnification of the mosquito proventriculus that shows a strong binding of FH to the epithelial surface (white arrows). (F) A higher magnification of the anterior and the anterior-posterior midgut that shows binding of FH to the epithelial surface (white arrows for the anterior midgut and red arrows for the anterior-posterior midgut). Dashed line in H shows the location of the sphincter between the foregut and midgut. (D and E) ACs of mosquitoes fed on sugar and blood, respectively. Solid squares show the location of the proventriculus. The red and yellow scale bars are 300 and 50 μm, respectively.

**Fig 4:**
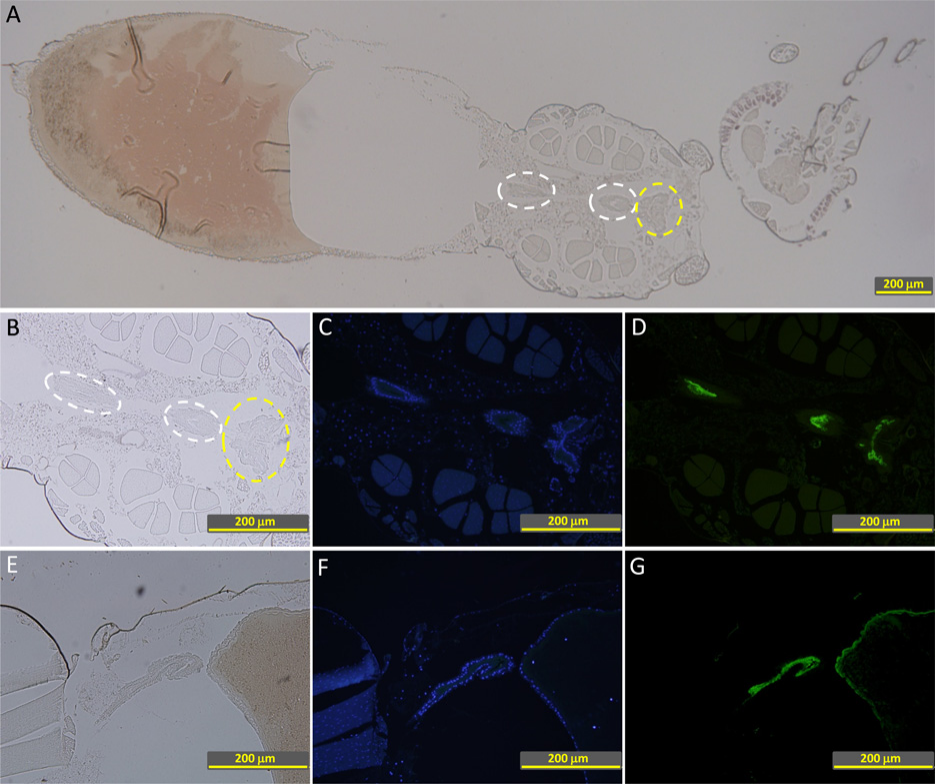
Binding of human FH to the *A. stephensi* mosquito proventriculus and midgut epithelial surface in indirect immunofluorescence assays. Female *A. stephensi* mosquitoes were fed with blood on a human volunteer’s arm and cut thereafter into coronal (A, B, C and D) or sagittal (E, G and F) sections. (A) Bright field microscopic image of whole mosquito coronal section showing the proventriculus (delineated by yellow dotted oval) and parts of the anterior midgut (delineated by white dotted ovals). (D) Binding of FH from the blood meal to the epithelial surface of the proventriculus and anterior midgut of the mosquito is shown as a green fluorescence. (G) Binding of FH from the blood meal to the epithelial surface of the anterior and posterior midgut is shown as a green fluorescence. (B-C and E-F) Bright field and DAPI nuclear staining images corresponding to D and G, respectively.

**Fig 5:**
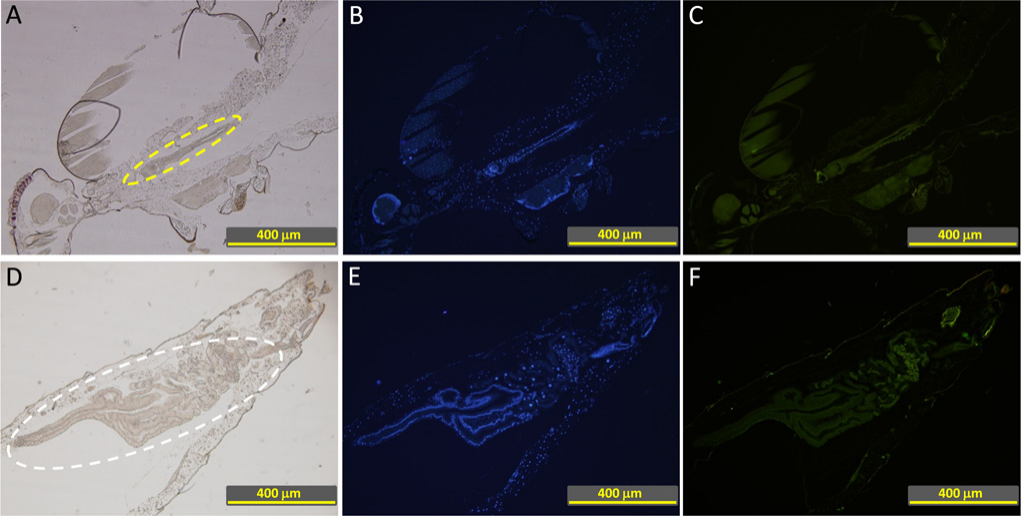
Control immunofluorescence assays using sugar-fed mosquitoes. (A and D) Bright filed microscopic images of *A. stephensi* mosquito sagittal sections of thorax and abdomen body parts, respectively, showing the proventriculus and the anterior midgut (delineated by yellow oval) and the posterior midgut (delineated by white oval) of the mosquito. (C and F) Anti-human FH did not react with any mosquito proteins as revealed by the absence of green fluorescence. (B and E) DAPI nuclear staining images corresponding to C and F, respectively.

**Fig 6:**
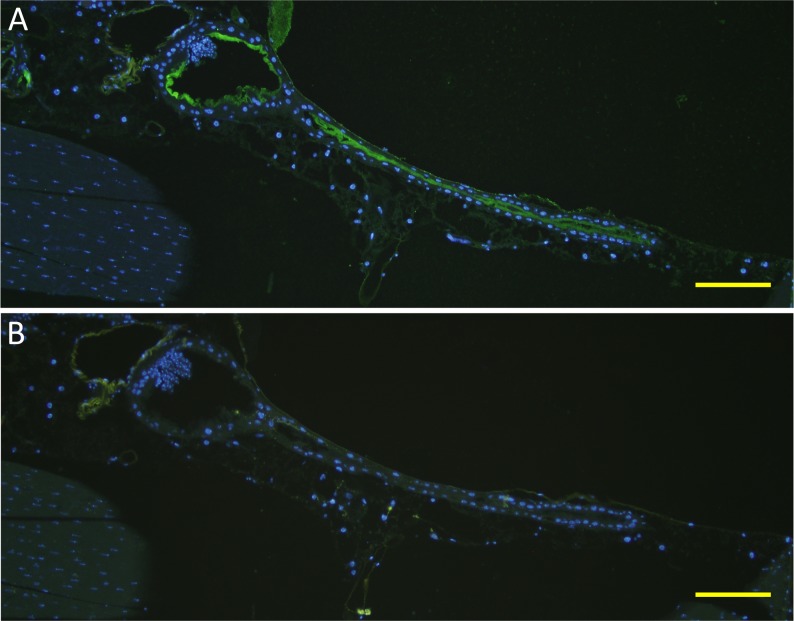
Binding of mouse FH to *A. stephensi* mosquito proventriculus and anterior midgut epithelial surface in indirect immunofluorescence assays. Female *A. stephensi* mosquitoes were fed on mouse blood using the membrane glass feeder and were cut thereafter into sections. (A) An overlay image showing binding of mouse FH from the blood meal to the epithelial surface of the proventriculus (oval shaped region) and anterior midgut (narrow tube region) of the mosquito as a green fluorescence and DAPI nuclear staining as a blue fluorescence. (B) An overlay image of a consecutive mosquito section probed with Alexa Fluor 488 Donkey anti-goat IgG only to control for cross reactivity towards mosquito and blood proteins in the absence of anti-FH. Absence of green fluorescence in (B) indicated lack of cross reactivity. Scale bars are 50 μm.

In our previous experiments complement in the mosquito midgut appeared to drastically reduce the bacterial load in the midgut PBF. Mosquitoes’ mortality as a likely indicator of midgut damage does not vary upon feeding on a blood meal containing functional or non-functional complement (common laboratory observation) Thus, we hypothesized that mosquito midgut epithelium has developed evasion mechanisms to escape the deleterious effect of complement activation. Acquisition of natural soluble complement regulators (Cregs) from blood by microbial surfaces is one of the most common mechanisms of complement evasion[12]. A similar mechanism that involves acquisition of Cregs by midgut epithelial surface could also occur when midgut is full of host blood. We tested this hypothesis by feeding *A. gambiae* and *A. stephensi* mosquitoes naturally on a human volunteer arm and looking for binding of Cregs and other complement components to mosquito gut epithelium. Sagittal sections of BF *A. gambiae* (Fig. 3A and 3F) and *A. stephensi* (Fig. 4E, 4G and 4F) mosquitoes were made 2 h post feeding. Coronal sections of *A. stephensi* (Fig. 4A, 4B, 4C and 4D) were also prepared. Immunofluorescence staining with antibodies against human FH, the soluble regulator of the alternative pathway of complement activation, revealed binding of FH to the epithelium surface of the proventriculus (Fig. 3B and 3C), the anterior midgut (Fig. 3G and 3H) and the anterior-posterior midgut (Fig. 3G and 3H). Sections made from another blood-fed *Anopheles* mosquito species, *A. stephensi*, showed identical results (Fig. 4) indicating that this phenomenon is not species-specific. The specificity of the anti-FH binding to its target was verified by immunofluorescence assays using sections of sugar-fed *A. stephensi* mosquitoes that showed no binding of anti-FH to mosquito proteins (Fig. 5). Additionally, binding specificity of the secondary antibody, Alexa Fluor 488 Donkey anti-goat IgG, was also verified in using sections of blood-fed *A. stephensi* mosquitoes in the absence of goat anti-FH from the IFA assays (Fig. 6B).

Parallel immunofluorescence assays did not show any sign of deposition of C3, C5 or MAC on the epithelial surface of the mosquito’s midgut suggesting protection from complement attack (S1 Fig).

**Fig 7:**
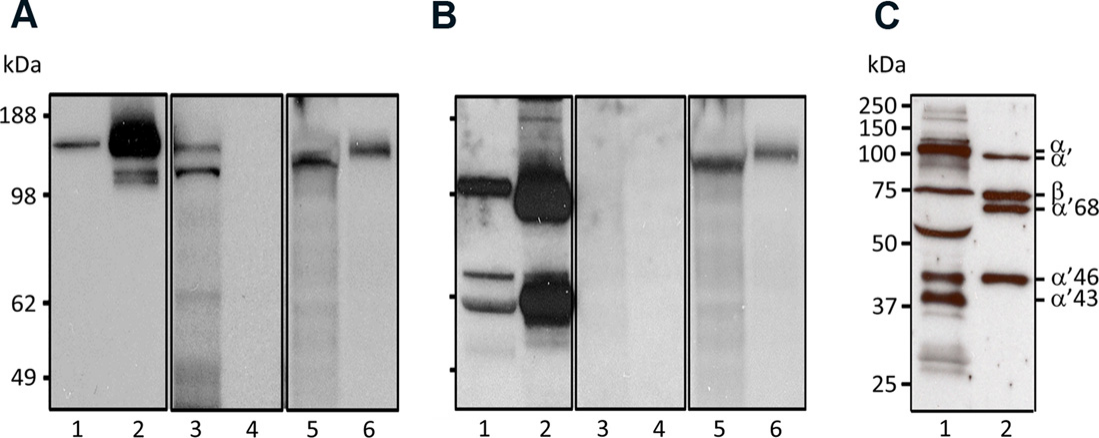
Western blots showing (A) binding of FH to *A. stephensi* mosquito alimentary canal, (B) the absence of C3 deposition on the alimentary canal and (C) inactivation of C3b in the blood bolus inside the midgut. Total protein extracts of ACs were isolated from blood (BF) and sugar-fed (SF) mosquitoes 2 h post blood feeding and analyzed by Western blotting. Panel (A) shows FH band (150 kDa) associated only with AC proteins from mosquitoes that were fed on blood (lane 3) but not with those fed on sugar (lane 4) and panel (B) shows the absence of C3 activation products in both BF (lane 3) and SF (lane 4) preparations. NHS diluted 1:400 was used as a positive control for anti-FH (A, lane 1)and anti-C3 (B, lane 1) binding to their targets. (C) Serum sample prepared from midgut content (blood bolus, lane 1) 2 h PBF showing FH+FI-mediated degradation of C3b represented by the 43 and 46 kDa bands. Bands representing the α chain of C3 and the α’ chain of C3b were also seen indicating incomplete activation of C3 and incomplete inactivation of C3b. A positive control lane was included to show the co-factor activity of FH in FI-mediated degradation of C3b in-vitro (lane 2). Lane 2 in A and B shows binding of anti-FH and anti-C3 to purified FH and C3b, respectively. Lanes 5 and 6 in A and B were probed with the MRA-258 monoclonal antibody to control for equal loading in lanes 3 and 4 in A and B.

To further confirm binding of FH to the mosquito midgut epithelium *A. stephensi* mosquitoes were allowed to feed on a volunteer arm and their ACs were dissected 2 h PBF. After washing the ACs SDS-soluble material was run on SDS-PAGE gel for detecting bound complement proteins by Western blot (WB) analysis. In agreement with the immunofluorescence microscopy observations FH was detected (Fig. 7A) among the proteins that were eluted from the AC tissues of BF mosquitoes. In contrast, anti-FH antibody did not detect any proteins in the eluted material from the midguts of SF mosquitoes. Furthermore, no signs of deposition of C3b or its inactivation products on the AC epithelium (Fig. 7B) were observed, when the same preparations were used in WB analysis and probed with an antibody that detects human C3, C3b, iC3b and C3c. The functional activity of FH in the mosquito midgut was also assessed by analyzing the soluble content of the midguts of BF mosquitoes. WB analysis revealed FH-dependent degradation of C3b by FI into iC3b (Fig. 7C) in the midgut content that was collected 2 h PBF. iC3b was apparent by the presence of 46- and 43-kDa fragment bands of the processed α’ chain of C3b. The WB analysis also showed that the 68-kDa fragment of iC3b (Fig. 7C, lane 2) was completely cleaved into smaller fragments (Fig. 7C, lane 1) suggesting either further cleavage of iC3b to C3c+C3d,g by erythrocyte CR1 and factor I or the involvement of midgut proteolytic activity in the complement inactivation process in the midgut.

Altogether, our data indicates that the surfaces of the proventriculus and the midgut capture FH to inhibit C3 deposition that otherwise could lead to lysis of the target cells. Moreover, complement activation in the soluble phase in the blood bolus seemed to be efficiently controlled by factor I-mediated C3b degradation and midgut proteolytic activity.

### Binding of mouse FH to mosquito midgut epithelium

Mouse serum containing active complement was also used to blood-feed *A. stephensi* mosquitoes to test for the ability of midgut epithelium to capture mouse FH from serum. Interestingly, immunofluorescence assays using goat anti-human FH that cross-reacts with mouse FH detected mouse FH on the surface of the midgut epithelium (Fig. 6A). FH signal was also more profound on the epithelium of the proventriculus and the anterior midgut as in the case of human FH binding to *A. gambiae* and *A. stephensi* midgut anterior midgut epithelium. Altogether, these data suggest that capturing FH by mosquito midgut epithelium from a blood meal could be a common mechanism utilized by blood-feeding mosquito species regardless of the host species.

### Human FH binding localizes to the apical surface of mosquito midgut epithelium

**Fig 8:**
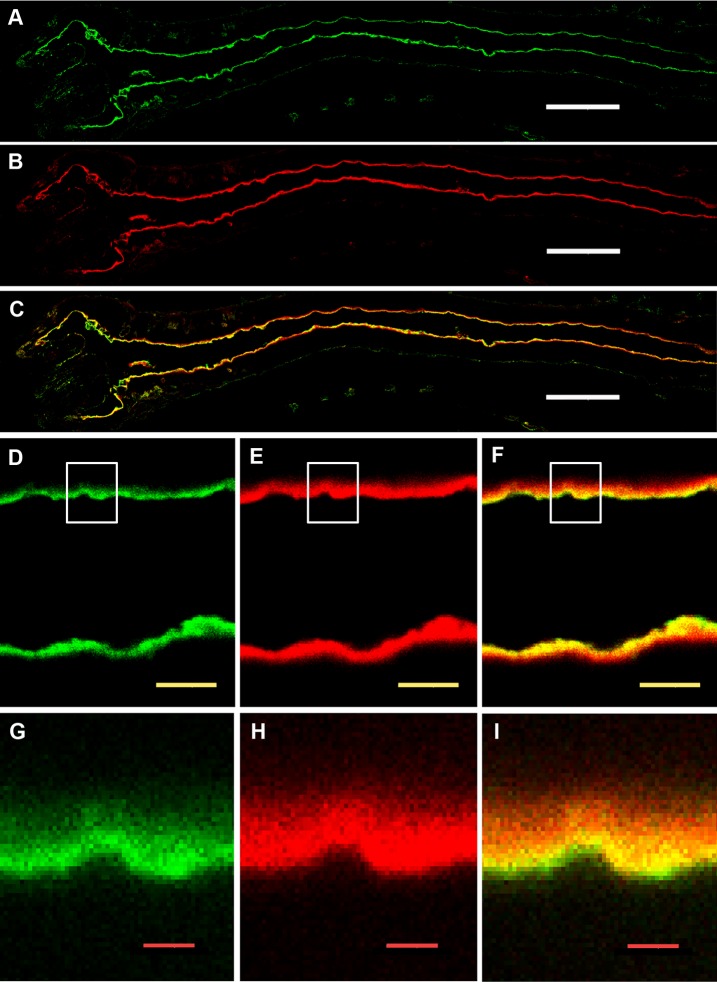
Confocal microscopy images showing colocalization of bound human FH with the glycocalyx layer at the apical surface of the proventriculus and the anterior midgut epithelium of *A. stephensi* mosquitoes. (A, D and G) FH binding to the surface of mosquito the proventriculus and the anterior midgut epithelium detected by goat Anti-FH. (B, E and H) Binding of ConA-Alexa 594 to the glycocalyx at the apical surface of the proventriculus and the anterior midgut epithelium. (C, F and I) Overlay images showing colocalization bound FH with the glycocalyx layer defined by bound ConA-Alexa 594. FH was found to colocalize with approximately one quarter of the luminal side of the glycocalyx layer (I). FH binding also formed a thin layer lining the luminal surface of the glycocalyx (C, F and I). The white, yellow and red scale bars are 50, 5 and 1 μm, respectively. G, H and I are digitally enlarged cropped images of the selected areas on D, E and F, respectively.

To further characterize FH binding to the midgut epithelium, 2 h PBF *A. stephensi* mosquito sections were double stained for FH and Concanavalin A (ConA) binding to the midgut surface. Confocal microscopy images showed a thick glycocalyx layer covering the outer surface of luminal plasma membrane represented by the red fluorescence of ConA-Alexa 594 (Fig. 8E and H). FH was found to colocalize with approximately one quarter of the luminal side of the glycocalyx layer (Fig. 8I). FH binding also formed a thin layer lining the luminal surface of the glycocalyx (Fig. 8C, F and I). Most of bound FH also appeared to colocalize with the glyococalyx. This data shows the association of FH binding to the mosquito midgut glycocalyx layer confirming binding of FH to a protein or a glycoprotein integrated into the plasma membranes of midgut epithelial cells.

### Detrimental effect of blocking FH activity on mosquito survival and fecundity

**Fig 9:**
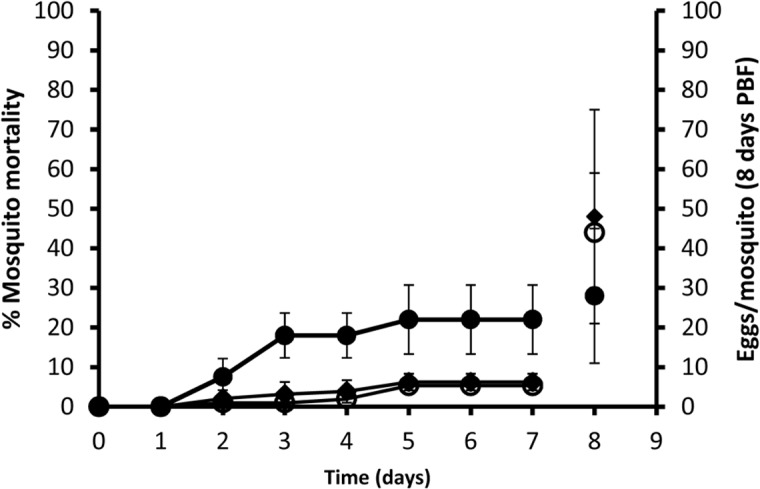
Effect of active serum with anti-FH on the *A. stephensi* mosquito survival and fecundity. Survival (left axis) and fecundity (right axis) of mosquitoes fed on a blood meal that contained NHS (**○**) or the monoclonal anti-human FH antibody (131X; 1 ug/ml) and either NHS (●) or HIS (♦). Mosquito survival was followed for 7 days post blood feeding and mosquitoes were allowed to lay eggs on day 8. Blocking FH activity with the 131X monoclonal antibody in the presence of NHS resulted in a significance increase in mosquito mortality and a decrease in egg count.

**Fig 10:**
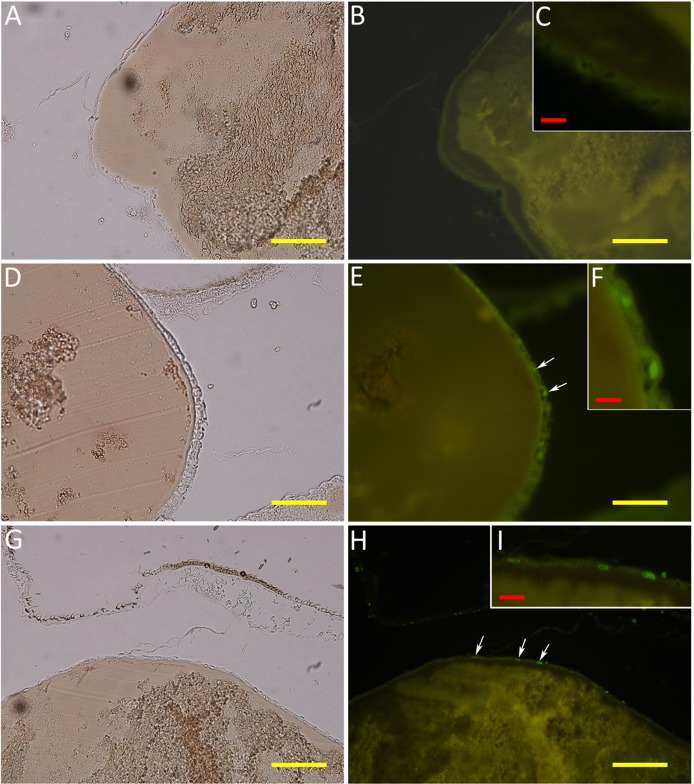
Detection of complement-mediated cell death induced by blocking of FH activity with the 131X monoclonal antibody in the midgut of blood-fed *A. stephensi* mosquitoes using the TUNEL analysis. (B and C) Mosquito sections showing absence of cell death in the posterior midgut epithelium of mosquitoes fed on blood containing NHS. (E and F) Mosquito sections showing injured cells induced after mosquito feeding on blood containing NHS and 131X antibody that blocks the protective activity of FH. (H and I) Apoptosis induced in-vitro on sections of mosquitoes fed on blood containing NHS as a positive control for the TUNEL analysis. (A, D and G) Bright-field images corresponding to B, E and H, respectively. White arrows point at the nuclei of apoptotic cells that are also enlarged in the insets F and I.

Binding of FH to the epithelial surface of the various compartments of the mosquito AC and the absence of C3b deposition prompted us to ask whether interfering with this binding would result in detrimental consequences. Therefore, *A. stephensi* mosquitoes were fed on blood meals that contained NHS alone or the monoclonal antibody, 131X, which functionally inactivates FH and either NHS or HIS. Mosquito mortality was first recorded at 48 h PBF and increased to 7% in the NHS/anti-FH-fed mosquitoes as compared with 1% and 2% in the NHS- and HIS/anti-FH-fed mosquitoes, respectively (Fig. 9). Mortality increased to 18% and 3% within 72 h in NHS/anti-FH- and HIS/anti-FH-fed mosquitoes, respectively. Mortality remained unchanged in the case of NHS-fed mosquitoes at the same time point. Highest mortality rates were observed at day 5 PBF in NHS/anti-FH, NHS- and HIS/anti-FH-fed mosquitoes being 22%, 5% and 6%, respectively (Fig. 9). No increase in mortality was reported until the last observation at day 7. The overall mortality was significantly different (Kaplan-Meier log-rank test, p<0.001) between NHS/anti-FH-and either NHS- or HIS/anti-FH-fed mosquitoes. To determine the effect of blocking FH activity in the midgut on mosquito fecundity, mosquito eggs were collected at day 8 PBF from NHS-, NHS/anti-FH- and HIS/anti-FH-fed surviving mosquitoes. The average number of eggs per mosquito was reduced to 28±17 eggs in the NHS/anti-FH-fed mosquitoes as compared with 44±15 and 48±27 eggs in the NHS- and HIS/anti-FH-fed mosquitoes, respectively (Fig. 9). From a subsequent identical experiment sagittal sections of mosquitoes were prepared 2 h PBF. Sections from six mosquitoes were then analyzed for the presence of apoptotic cells as a result of neutralizing FH with the 131X monoclonal antibody. TUNEL assays showed signs of cell death presented as green fluorescent nuclei (Fig. 10E and F) in midgut epithelium in one mosquito out of six in the 131X-neutralized FH group (131X+NHS group). No signs of cell death were detected in the absence of the 131X anti-FH antibody in sections of seven mosquitoes (Fig. 10B and C) comprising the control group (NHS group). Altogether, these results suggest that blocking FH-mediated complement evasion in the mosquitos has deleterious effect on the mosquito’s survival and the fecundity of those mosquitoes that managed to survive complement-mediated damage.

### Binding of FH to 40 and 100 kDa proteins in the mosquito midgut

**Fig 11:**
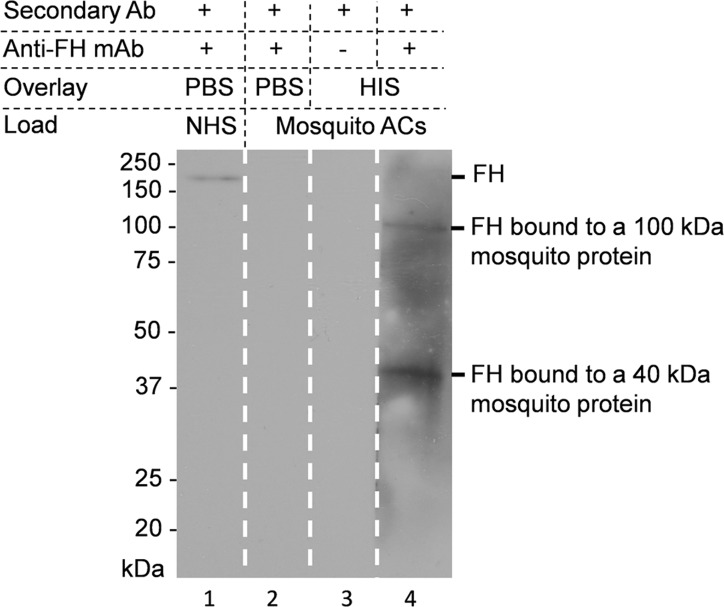
Ligand blot assay showing binding of FH to *A. stephensi* mosquito AC proteins. AC proteins were prepared from mosquitoes and run on non-reducing SDS-PAGE gels (lanes 2–4). After transfer to a nitrocellulose sheet the lanes were overlaid with PBS (lane 2; control) or HIS (lanes 3 and 4) as indicated. Lane 4 shows binding of FH to 40 and 100 kDa mosquito AC proteins. No reaction was detected when anti-FH was replaced by PBS-T in control lane 3, respectively. The reactivity of the monoclonal anti-human FH antibody (131X) against native FH in NHS was confirmed in lane 1.

To shed light on the interaction of FH with mosquito midgut proteins, we initiated a search for the potential FH receptor(s). The ACs of *A. stephensi* mosquitoes that had never fed on blood were dissected and membrane proteins were extracted. The extracted proteins were run onto SDS-PAGE gel under non-reducing conditions and transferred to nitrocellulose sheets for ligand blot analysis to detect binding of FH to mosquito proteins in vitro. As shown in Fig. 11 two mosquito proteins of approximately 40 and 100 kDa were found to be the candidate receptors for binding FH.

## Discussion

In this study we observed binding of the major complement inhibitor factor H to the alimentary canal epithelium of the *Anopheles* mosquitoes. Apparently, this interaction protects the mosquito’s epithelium from complement-mediated damage and could provide a target for a transmission blocking vaccine-induced immunity. On the other hand, we found that complement becomes activated in the fluid phase in the mosquito midgut. As a consequence, complement activation was able to significantly reduce the number of the bystander bacteria in the midgut, a phenomenon that could help *Plasmodium* parasites to survive in the mosquito midgut. Further experiments showed that blocking of FH activity by a monoclonal antibody that interferes with its function and boosts complement activation had a detrimental effect on mosquito survival and fecundity.

Earlier studies, with a particular interest in the resistance of *Plasmodium* gametes to complement activity, reported long-lasting persistence of complement components or activity in the mosquito midgut post blood feeding. Margos et al [13] observed that rat complement components necessary to initiate the alternative pathway (factor B, factor D, and C3) as well as C5 were present for several hours following the ingestion of *P. berghei*-infected rat blood. In a more recent study, Simon et al [14] have shown that the concentration (OD values) of C3a, a marker for complement activation, was 2.3-fold higher in mosquito midgut at 1 h PBF (artificial feeding) compared to control. In the current study, mosquito’s natural feeding on a human volunteer arm showed that complement became strongly activated in the mosquito midgut. C3a concentration (ng/ml) was 100-fold higher within 10 min PBF and dropped to 7-fold at 30 min PBF compared to the basal level in the human volunteer serum. The sharp decrease in C3a level was probably due to its rapid binding to receptors and/or to anionic surfaces because of its cationic nature. Leukocytes present in the blood meal carry receptors for C3a (C3aR). SC5b-9 is a more stable complement activation product than C3a and the result of the full activation sequence. Therefore, the SC5b-9 level peaked after the C3a peak, as expected from the kinetics of complement activation. This was followed by a slight decrease in the SC5b-9 level indicating that no more activation was taking place at 90 min PBF. The presence of a considerable amount of C3 in the mosquito midgut at 90 minutes PBF suggested that some C3 activity was still left. The residual activity assays, however, showed complete loss of the alternative pathway activity at 10 min PBF, whereas, about 20% of the classical pathway activity still remained at 90 min PBF in an assay format that detects the ability to generate SC5b-9 complexes. This finding suggested that the classical and the terminal pathway components were partially active and functional C3 was involved in the measured residual activity. In contrast, in the alternative pathway a rate limiting factor was consumed or, alternatively, the pathway had become specifically inhibited. An earlier study showed that soluble molecules from the midgut of the mosquito *Aedes aegypti* inhibited complement activation and C3b deposition in vitro via the alternative pathway by 52% and via the classical pathway by only 24%[15]. Altogether, the current data suggests that some mechanisms of complement inhibition operate in the mosquito midgut, and that while complement becomes activated for some time in the fluid phase the mosquitoes do not seem to suffer from complement attack.

The potential ability of complement in the mosquito midgut to cause damage to cells was recognized from the significant decrease in the number of viable bacteria post mosquito feeding on NHS-supplemented blood as compared with feeding on HIS-supplemented blood. Complement-mediated killing of the midgut bacteria was taken as an indirect indication that this could have been the fate of the midgut epithelium unless there was an evasion mechanism that allowed the epithelial cells to escape complement-mediated damage. Complement-mediated killing of midgut bacteria could also lead to consumption of complement components that could otherwise target the midgut epithelium. The lack of damage to mosquito cells was supported by the absence of nuclear chromatin fragmentation, a sign of cell death, in the midgut epithelial cells. In agreement with our postulation, a previous study in which the bug *Triatoma brasiliensis* was fed by the forced feeding procedure to bypass mixing blood with the saliva that contains a complement inhibitor showed significant signs of midgut cell death as early as 1 h PBF [15]. This finding supports a possible role for complement inhibitors of hematophagous vectors in protecting midguts from host complement-mediated injury. Additionally, mosquitoes fed on mice immunized with mosquito ACs homogenate have shown a significant reduction in mosquito survival compared to control groups [16]. A similar study has also shown impairment of the development of *Plasmodium* spp. inside the *Anopheles* spp. midgut and, thereby, a block in parasite transmission [17]. It is likely that mosquito killing in these studies was mediated by antibody-mediated activation of the classical complement pathway, for which less inhibitory activity was found to operate in the mosquito midgut in our study. On the other hand, antibodies neutralizing an inhibitor of the alternative pathway would enhance complement killing of the midgut cells. In addition to complement-mediated damage, mosquito killing could also be due to interference of anti-AC antibodies with vital cell functions on the mosquito midgut surface.

The observed drastic complement-mediated reduction of bacterial count in the mosquito midgut also argues for a special attention of studies focusing on understanding the association of midgut microbiota with the malaria parasite survival in the mosquito midgut [18,19]. Natural blood feeding could thus greatly reduce the number of midgut bacteria by complement-mediated killing, as opposed to artificial feeding that is usually based on heat-inactivated serum. Shaping the diversity of gut microflora of hematophagous species was also reported for the *Hirudo medicinalis*, a medicinal leech, as active complement in the ingested blood limited the gut microflora to only the complement-resistant bacterial strains[20].

Using an indirect immunofluorescence assay FH was detected to bind to the mosquito proventriculus and midgut. C3, C5 and MAC were absent from the ACs epithelial surface indicating the protective activity of the cell-bound FH. Factor H binding is thus a potential complement evasion mechanism developed by the mosquitoes to protect their ACs from complement-mediated damage. Furthermore, a monoclonal antibody directed against FH that is known to inhibit its function significantly increased the mortality rate in blood-fed mosquitoes within 72 h from about 1% in NHS-supplemented blood meal to 18% when NHS was supplemented with anti-FH. The relatively low level of mortality, albeit significant, in the presence of the anti-FH monoclonal antibody could have resulted from insufficient quantities of the monoclonal antibody, rapid consumption of active complement or mosquito inhibitors of complement activation. Complement can become spontaneously activated, and this process is accelerated in the presence of an anti-factor H antibody.

In our attempts to identify mosquito’s FH-binding molecules we confirmed this interaction by the ligand blotting assays and detected two molecules with molecular masses of about 100 and 40 kDa as potential FH binding molecules. The identities of these molecules are the subject of ongoing work. It is also possible that, in addition to specific proteins, mosquito cell surface glycosaminoglycans or other polyanions could play a role in FH binding to mosquito AC cell surfaces. Exploiting soluble complement regulators, particularly FH, from the host by many pathogenic bacteria has been well described [5]. Factor H binding has been reported also to fungi, viruses and parasites [5,14]. The current study is to our knowledge the first one to describe the utilization of a similar complement evasion strategy by a hematophagous vector. Previous reports on complement evasion or inhibition by hematophagous vectors were almost exclusively from studies on ticks. Those studies identified other unique strategies to block complement activation. For example, a salivary protein, OmCI, from the *Ornithodoros moubata* tick, the vector of human relapsing fever caused by *Borrelia duttoni*, was shown to specifically bind and inhibit C5, thereby preventing activation of the terminal complement pathway[21]. Another salivary protein, TSLPI from *Ixodes scapularis* tick, vector of the Lyme disease caused by *Borrelia burgdorferi*, was shown to interfere with the lectin pathway of complement activation by preventing MBL binding to its ligand[22]. TSLPI was also shown to be beneficial to *B. burgdorferi* as it resulted in impaired neutrophil phagocytosis and chemotaxis and reduced killing of *Borrelia* [22]. A third strategy that was shown to be utilized by ticks to block complement activation was binding of tick salivary proteins such as Isac, Irac-1 and-2, and Salp20 to properdin and displacing it from the alternative pathway C3 convertase resulting in its inhibition[23–25]. So far, no analogous anticomplement strategies have been identified in mosquitoes, except the one in the current study. However, mosquito antihemostatic molecules such as anophelin from *Anopheles* spp. [26,27], anti-factor Xa from *Aedes aegypti*[28] and alboserpin from *Aedes albopictus*[29] that target human coagulation system were already identified in mosquito’s saliva.

Molecules involved in complement inhibition from hematophagous vectors are of potential interest to generate an anti-vector vaccine that could interfere with the lifespan of the disease vectors or with infectivity of the pathogen. Studies to envisage the possible use of arthropod vector proteins as anti-vector vaccine already exist[30], but none of them have attempted to use a complement regulator-neutralizing vaccine so far. Thus, mosquito midgut antigens involved in essential biological processes such as in complement inhibition would serve as more specific candidates for similar studies. Moreover, identifying complement inhibitors from hematophagous vectors could also provide pharmacological agents to treat diseases, where complement activation is known to play a role[31].

In conclusion, we have shown in this study that the complement system becomes immediately activated in the mosquito after ingestion of human blood while, at the same time, the mosquito AC surface molecules captured FH from the blood meal and inhibited the deposition of C3b on the midgut epithelium. The initial complement activation that occurred in the blood bolus in the midgut was able to kill midgut bacteria that were not resistant to complement. On the other hand, acquisition of FH by the midgut epithelial cells contributed to mosquito’s survival against the innate immune system in the ingested blood meal. Interfering with the complement regulatory activity of FH in the mosquito midgut increased mosquito mortality and reduced fecundity. The putative *Anopheles* mosquito FH binding proteins could be transmission blocking vaccine candidates targeting the malaria parasite carrying vectors.

## Supporting Information

S1 Fig Binding of C3, C5 and MAC were assessed on sagittal sections made 2h post blood feeding of *A. gambiae* mosquitoes fed on a human volunteer arm. C3, C5 and MAC were absent from the surface of the posterior midgut epithelium, A, B and C, respectively.
(TIF)
