# Supplementary figures and images for: Anopheles Midgut Epithelium Evades Human Complement Activity by Capturing Factor H from the Blood Meal
Source: PLoS Negl Trop Dis. 2015 Feb 13;9(2):e0003513. doi: 10.1371/journal.pntd.0003513 (PMC4332473; doi:10.1371/journal.pntd.0003513)

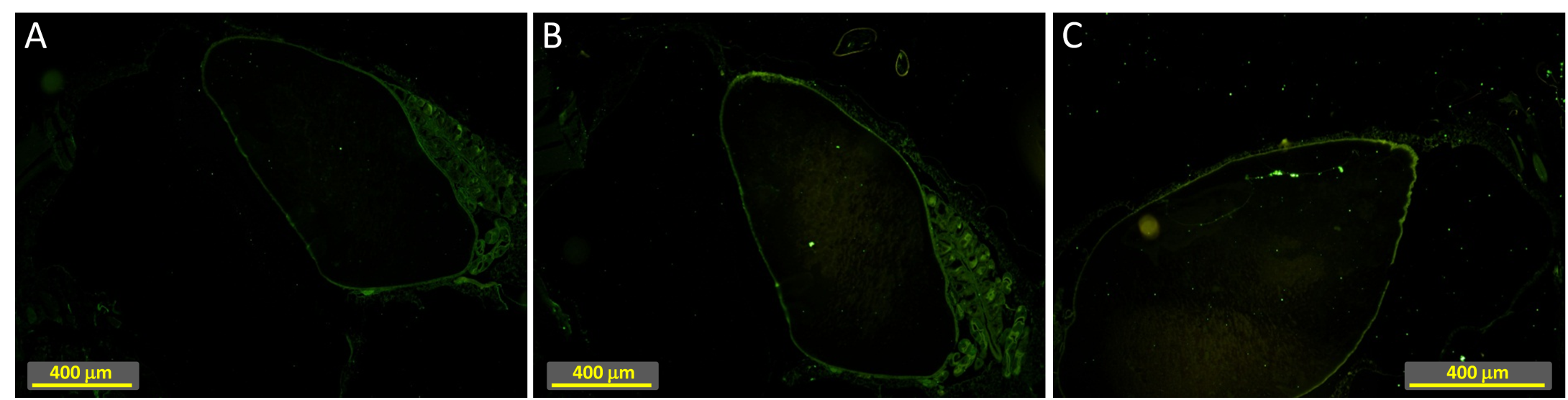

Supplement: S1 Fig — C3, C5 and MAC were absent from the surface of the posterior midgut epithelium, A, B and C, respectively. (TIF) [file pntd.0003513.s001.tif]
